# Efficacy of Oral Midazolam for Sedation and Amnesia in Preschool Children with Dental Anxiety: A Double-Blind, Randomized Controlled Trial

**DOI:** 10.3390/dj13070308

**Published:** 2025-07-09

**Authors:** Nguyen Quang Binh, Vo Truong Nhu Ngoc, Pham Quoc Khanh, Nguyen Phuong Huyen, Trinh Khanh Linh, Pham Phuc Khanh, Le Kha Anh

**Affiliations:** 1 National Hospital of Odonto-Stomatology, Hanoi 100000, Vietnam; nguyenbinh3010@gmail.com (N.Q.B.); huyenrangtreem@gmail.com (N.P.H.); 2 School of Dentistry, Hanoi Medical University, Hanoi 100000, Vietnam; votruongnhungoc@gmail.com (V.T.N.N.); linh120903@gmail.com (T.K.L.); lekhaanh268@gmail.com (L.K.A.); 3 National Children’s Hospital, Hanoi 100000, Vietnam; 4 Department of Otorhinolaryngology, University of Medicine and Pharmacy-Vietnam National University, Hanoi 100000, Vietnam; dr.khanhpham.ent@gmail.com; 5 Division of Research and Treatment for Oral Maxillofacial Congenital Anomalies, Aichi Gakuin University, Nagoya 464-8651, Japan

**Keywords:** amnesia, dental anxiety, oral midazolam, sedation, pediatric dentistry

## Abstract

**Background**: Dental anxiety often poses a significant barrier to effective dental care in pediatric patients. This study evaluated the efficacy and safety of oral midazolam at two different doses for sedation and amnesia in preschool children undergoing dental procedures under 30 min, including primary teeth extraction, root canal treatment, dental filling, and stainless-steel crown. **Methods**: This prospective, double-blind, randomized controlled trial included 80 children aged 3–6 years with dental anxiety (Types 1 and 2 of the Frankl Behavior Rating Scale) at the National Hospital of Odonto-Stomatology, Hanoi. following the CONSORT guidelines. Participants were randomly assigned to receive oral midazolam at 0.3 mg/kg or 0.6 mg/kg. Sedation efficacy, onset time, procedure duration, cooperation level (Houpt Behavior Rating Scale), recovery time, and amnesia at 12 h, 24 h, and 1 week, as well as adverse events, were recorded and analyzed. **Results**: Both midazolam doses were effective for sedation (0.3 mg/kg: 95% vs. 0.6 mg/kg: 100%, *p* = 0.49). The higher dose (0.6 mg/kg) provided significantly longer effective procedural time (27.3 ± 4.1 min vs. 20.3 ± 4.0 min, *p* < 0.001) and better patient cooperation (95% vs. 78.9%, *p* = 0.045), but slightly prolonged recovery time (34.6 ± 4.6 min vs. 31.8 ± 4.4 min, *p* = 0.008). Both doses produced similar amnesic effects, with approximately 90% anterograde amnesia at 12 h post-procedure. Adverse events were minimal and mild. **Conclusions**: Both 0.3 mg/kg and 0.6 mg/kg doses of oral midazolam are safe and effective for sedation and amnesia in preschool children undergoing dental procedures. While the 0.6 mg/kg dose provides better procedural cooperation and prolonged sedation, it also requires a slightly longer recovery time.

## 1. Introduction

Dental anxiety is a common concern in pediatric dentistry, especially among preschool-aged children. Research estimates its prevalence at around 36.5%, with some studies reporting rates as high as 70% in certain populations [1]. Dental anxiety often leads to the avoidance of necessary care, increasing the risk of untreated caries and poorer oral health outcomes. Studies show that children with dental anxiety are more prone to severe dental disease, which can negatively affect their family’s overall quality of life [2].

Behavioral management techniques, such as positive reinforcement and distraction, are commonly used to reduce anxiety. However, they may be insufficient for children with severe anxiety or uncooperative behavior. In such cases, pharmacological sedation is a well-established approach that facilitates treatment while ensuring a more positive experience for both the patient and the practitioner [3].

Midazolam, a short-acting benzodiazepine, is frequently used in pediatric sedation due to its anxiolytic, sedative, and amnesic properties. It enhances the activity of gamma-aminobutyric acid (GABA) at GABA-A receptors, resulting in central nervous system depression, which induces relaxation and reduces memory retention of distressing experiences. These characteristics make midazolam a preferred agent for pediatric dental sedation, as it improves cooperation and minimizes traumatic memories [4]. Midazolam can be administered via intravenous (IV), intranasal, and oral routes. Although IV midazolam is associated with rapid onset and reliable bioavailability, its use in young children is often limited due to the need for venous access, which can induce anxiety and distress. Conversely, oral administration is non-invasive, well-tolerated, and widely preferred in pediatric dentistry despite its slower onset and variable bioavailability [5,6]. Current clinical guidelines recommend oral midazolam doses ranging from 0.25 to 1 mg/kg, depending on the patient’s status and desired effect. The 0.3 mg/kg dose is widely used and has shown effective sedation with minimal side effects, making it a common choice in clinical settings [7]. However, some studies suggest that a higher dose, such as 0.6 mg/kg, may enhance sedation success, particularly in uncooperative children or procedures requiring deeper sedation [8,9,10,11]. Therefore, further research is needed to determine the optimal dosing strategy for balancing efficacy and safety in pediatric patients.

Amnesia plays a critical role in pediatric dental treatment by reducing memory recall of distressing experiences, which may help alleviate anxiety in future dental visits and improve long-term compliance with oral healthcare [10]. However, despite the extensive evidence supporting midazolam-induced amnesia, there remains limited research specifically comparing the efficacy of different oral doses in pediatric dentistry. Understanding the optimal dose that balances effective sedation with adequate amnesia is essential for improving patient experience and procedural success.

This study aimed to compare the sedative and amnesic effects of different oral midazolam doses in preschool children with dental anxiety. Additionally, we evaluated the safety profiles of doses by monitoring vital signs and adverse events.

## 2. Materials and Methods

### 2.1. Study Design

This prospective, double-blind, randomized controlled trial (RCT) was conducted from 2022 to 2024 following the CONSORT guidelines at the National Hospital of Odonto-Stomatology and the National Children’s Hospital in Hanoi, Vietnam. The study received ethical approval from the Institutional Review Board (IRB) of Hanoi Medical University (IRB-VN1.001/IRB00003121/FWA 00004148) under number 1570 and adhered to the ethical principles of the Declaration of Helsinki. Informed consent was obtained from all participants’ parents or legal guardians after they were fully informed about the study’s objectives, procedures, potential risks, and benefits, ensuring respect for their rights, dignity, and welfare. This study is registered in ClinicalTrials.gov under the registration number (NCT06887712-1 April 2024).

### 2.2. Participants

The study included 80 participants of children aged 3 to 6 years who were presented with dental anxiety at the National Hospital of Odonto-Stomatology, Hanoi, Vietnam. The sample size of the study was calculated using the formula for determining sample size in a randomized controlled clinical trial, based on previous studies [11,12]. The minimum required sample size for this study was 78 participants. However, in practice, we enrolled 80 patients. The inclusion criteria were (1) children aged 3 to 6 years old; (2) with dental anxiety categorized as “definitely negative” or “negative” on the Frankl Behavior Rating Scale [13], as determined by a specialist in pediatric dentistry; (3) within the normal range of weight; and (4) classified as ASA I or ASA II according to the American Society of Anesthesiologists (ASA) physical status classification. Exclusion criteria included the following: (1) children with diseases of the endocrine system, severe respiratory conditions, heart diseases, mental health conditions (such as autism, ADHD, or schizophrenia), abnormal brain development or cognitive impairments; (2) children with abnormal liver function or abnormal kidney function; and (3) children with known allergies to any drugs used in the study.

### 2.3. Randomization and Blinding

Due to the study’s design and the inability to recruit all 80 participants at once, block randomization was used to assign participants to one of two groups. Each time two eligible patients consented to participate in the study, they were randomly assigned to one of the two groups for the study in a pairwise manner. The dentist performing the dental procedure and the data collectors were blinded to the doses assigned.

The principal investigator prepared two sealed envelopes. Each envelope contained a piece of paper with a number written on it: “1” corresponded to the 0.3 mg/kg dose of oral midazolam, and “2” corresponded to the 0.6 mg/kg dose. The anesthesiologist responsible for sedation, who was aware of the labeling system, randomly selected one envelope for the first patient and administered the corresponding dose. The second patient received the remaining dose from the other envelope. After the doses were administered, the anesthesiologist was not involved in data collection or analysis. The dentist performing the dental procedure was blinded to the doses assigned. The data collectors were aware only of whether the child belonged to Group 1 or Group 2, but were not informed of the labeling system or the specific dose corresponding to each group.

### 2.4. Study Procedures

A standard preoperative fasting period of 6 h for solids and 4 h for liquids was followed to ensure gastric emptying before sedation.

Before sedation (T_0_), baseline vital signs, including heart rate and peripheral oxygen saturation (SpO_2_), were monitored and recorded. Each child received the assigned dose of oral midazolam (0.3 mg/kg or 0.6 mg/kg). Since the oral form of midazolam is not commercially available in Vietnam, the intravenous formulation Midazolam Braun 5 mg/mL (B. Braun Melsungen AG, Hessen, Germany) was diluted and mixed with orange juice to create an oral solution. The midazolam dose was administered orally in the anesthesia area.

The level of sedation was assessed using a 6-point Ramsay sedation scale (RSS) (Table S1) [14] by a qualified anesthesiologist. Once the child reached a score of 3, they were separated from their parents and taken to the dental procedure room.

Successful sedation was defined as the child reaching a score of 3 on the RSS within 30 min. Most of the children who come for examination and treatment at our hospital exhibit a high level of uncooperativeness and have previously undergone unsuccessful interventions at dental clinics. Since most of the procedures involve pain, it is necessary to achieve a Ramsay sedation score of 3. If the child does not reach an RSS score 3 after 30 min, they will be considered as having unsuccessful sedation and no further data will be recorded for the study. Sedation onset time was defined as the interval from the administration of oral midazolam to the moment the child achieved a score of 3 on the RSS.

The dental procedure was expected to be completed within 30 min, including primary teeth extraction, root canal treatment, dental filling, and stainless-steel crown. The dental procedure began once the child was sedated, and local anesthesia (2% lidocaine with 1:80,000 epinephrine) was administered if needed for the dental treatment. Throughout the dental procedure, vital signs were continuously monitored and recorded immediately after midazolam administration (T1), 5 min later (T2), 10 min later (T3), 20 min later (T4), and post-dental procedure (T5).

The dental procedure time referred to the time spent by the dentist performing the procedure on the child, calculated in two different scenarios: (1) In cases where the child cooperated fully from start to finish (from Excellent to Fair), it was the total treatment time. (2) In cases where the child initially cooperated well but later showed signs of non-cooperation, making it impossible to continue (Poor), it was calculated from the start of the treatment until the treatment was interrupted.

After the procedure, the child was reunited with their parents and waited in the recovery room. They were allowed to have light food and drink after 10–15 min. The child was then discharged from the hospital after being assessed and meeting the criteria (9 or 10 points) according to the Post-Anesthetic Discharge Scoring System (PADSS) [15]. Recovery time was calculated from the end of the dental procedure until the child reached a PADSS score of ≥9.

### 2.5. Outcome

#### 2.5.1. During-Treatment Behavior Assessment

Behavior during treatment was assessed using the Houpt Behavior Rating Scale [16], which includes evaluations of sleepiness, movement, and crying behavior (Table S2). This process was independently assessed by two qualified pediatric dental specialists during the dental procedure. In case of any discrepancies, the results were further reviewed and discussed until a consensus was reached.

#### 2.5.2. Memory Assessment

To assess the amnesic effect of midazolam, children were asked to recall details of the procedure 12 h, 24 h, and 1 week after the treatment. The ability to remember aspects of the procedure was evaluated through a simple verbal questionnaire conducted via a video call by a researcher. The assessment questionnaire was designed for the parents of the children; parents asked their children questions and provided responses to the investigators. The reliability level was high, as the parents had been informed about the importance and details of providing accurate answers. The questionnaire contained 5 questions:

(1) “Did your child mention or say anything about the dental visit after coming home?”
(2) “Was your child able to describe any dental instruments (e.g., the light, saliva suction device) or sounds in the clinic?”
(3) ”When hearing the words ‘dentist’ or ‘dental doctor’, does your child show signs of anxiety or fear?”
(4) “Did your child complain of pain or discomfort in the teeth/mouth after the treatment?”
(5) “If a follow-up appointment is scheduled, does your child react positively (e.g., positively agrees) or negatively?”

#### 2.5.3. Safety Assessment

Adverse events were monitored during the procedure and for 24 h post-sedation. The primary safety outcomes included signs of respiratory depression, hypotension, nausea, vomiting, or excessive sedation. These events were recorded and analyzed to determine the safety profile of each dose.

### 2.6. Statistical Analysis

The data were normally distributed; therefore, parametric statistical tests were employed for analysis. The data were analyzed using SPSS Statistics for Windows, Version 20.0 (Armonk, NY, USA). Descriptive statistics, including mean and standard deviation (SD), were calculated for continuous variables such as age, weight, sedation onset time, dental procedure time, and recovery time. Frequencies and percentages were calculated for categorical variables such as sex, ASA classification, sedation efficacy, and amnesic effect.

Independent *t*-tests were used to compare continuous variables between the two groups (0.3 mg/kg and 0.6 mg/kg doses). Chi-square tests were applied to compare categorical variables such as sedation efficacy and amnesic effect, and Fisher’s exact test was used when more than 20% of the cells had expected values below 5.

The *p*-value for statistical significance was set at <0.05. All analyses were conducted at a two-tailed significance level.

## 3. Results

### 3.1. Demographic Data

The demographic characteristics of the participants are shown in Table 1. 80 children aged 3 to 6 years were included in the study, with 40 children in each group. The mean age was 4.7 ± 0.9 years in both groups. There was no significant difference between the groups regarding sex, ASA classification, or Frankl scale scores. The average weight of the children in the 0.3 mg/kg group was 16.45 ± 2.87 kg; in the 0.6 mg/kg group, it was 16.88 ± 3.49 kg. The differences in these variables were not statistically significant (*p* > 0.05).

### 3.2. Sedation Effects

**Figure 1:**
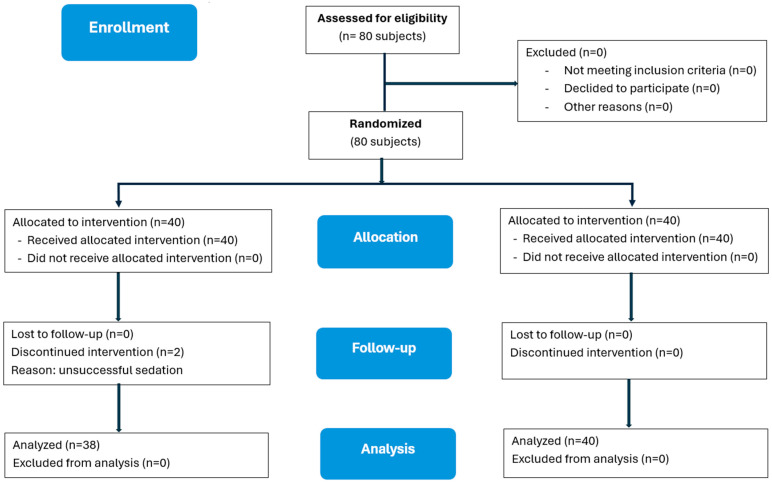
CONSORT flow diagram of the study.

As shown in Table 2, 95% of children in Group 1 and 100% in Group 2 achieved successful sedation, with no significant difference between the two groups (*p* = 0.49). Therefore, 78 patients were allocated to 2 groups (Figure 1). The sedation onset time was similar between the two groups, with 21.1 ± 3.1 min for Group 1 and 20.3 ± 3.1 min for Group 2 (*p* = 0.26).

Group 2 (0.6 mg/kg dose) offered significantly more time for the dental procedure, with an average procedure time of 27.3 ± 4.1 min compared to 20.3 ± 4.0 min in Group 1 (*p* < 0.001).

The recovery time was significantly longer in Group 2 (34.6 ± 4.6 min) compared to Group 1 (31.8 ± 4.4 min, *p* = 0.008).

### 3.3. Behavior During Treatment

During treatment, assessed using the Houpt Behavior Rating Scale, 95% of the children in Group 2 exhibited high cooperation (Excellent, Very good, Good, Fair), compared to 78.9% in Group 1 (*p* = 0.045). This difference was significantly different (Table 3).

### 3.4. Amnesic Effects

Both doses of oral midazolam produced similar amnesic effects, with no significant differences between the groups at the three time points (12 h, 24 h, and 1 week) (Table 4).

### 3.5. Safety Assessment

Table 5 presents vital signs for both groups. Heart rate and SpO_2_ levels were similar between the groups throughout the procedure. The heart rate remained stable in both groups, with slight decreases noted at later time points. Oxygen saturation (SpO_2_) remained within normal limits for all participants.

Adverse events were minimal, with no respiratory events or other severe side effects observed at either the 0.3 mg/kg or 0.6 mg/kg dose. Specifically, 5% of children in Group 1 experienced nausea, while 5% in Group 2 experienced agitation (Table 4).

## 4. Discussion

This study aimed to evaluate the sedative and amnesic effects of two oral doses of midazolam (0.3 mg/kg and 0.6 mg/kg) in preschool children with dental anxiety. The demographic characteristics, including age, gender, ASA classification, baseline anxiety, and weight, were similar between the two study groups, minimizing potential confounding effects related to patient demographics. The use of a randomized, double-blind study design ensured that biases related to treatment assignment and observer assessments were effectively controlled.

Previously, one study had evaluated the effectiveness of 0.3 and 0.5 mg/kg of oral midazolam in dental treatment in 20 patients with definitively negative behavior (Frankl Level 1) [17]. Another study was conducted in 2014 to compare 0.5 mg and 0.75 mg/kg with 23 patients [18]. This study compares doses of 0.3 and 0.6 mg/kg with a larger sample size of 80 patients (90% of whom were Frankl Level 1), which enhances the statistical power and reliability of the results. Moreover, this is the first study to comprehensively assess all three aspects: efficacy, safety, and amnesic effect in children aged 3 to 6 years after dental treatment.

### 4.1. Sedation Effects

Both doses of oral midazolam were effective in providing sedation, with 95% of children in the 0.3 mg/kg group and 100% in the 0.6 mg/kg group achieving successful sedation. Similarly, Wheeler’s study reported a sedation success rate of 93% with a 0.5 mg/kg dose [19], and Sultan Keles’ study found a slightly higher success rate of 96.2% using the same dosage [20]. Caution is needed when comparing sedation success rates across studies due to variations in study design, sedation scales, and observer interpretation. For example, the timing of assessment differs in some studies [20,21]. Sedation was assessed using a range of distinct sedation scales, with most studies employing 3- to 6-point scales, while a few utilized the Observer’s Assessment of Alertness/Sedation (OAA/S) scale, where effective sedation was defined as a score of 17 or less on a 20-point scale [7,22]. The current study adopted the Ramsay Sedation Scale due to its practicality and ease of use, allowing for quick and repeated assessments within short intervals. However, RSS may introduce greater subjectivity, as it relies heavily on observer interpretation.

The sedation onset time was slightly longer in the 0.3 mg/kg group compared to the 0.6 mg/kg group (21.1 ± 3.1 min vs. 20.3 ± 3.1 min), although this difference did not reach statistical significance (*p* = 0.26). Previous studies demonstrated that oral midazolam typically induces sedation within 15 to 30 min [9,18]. Additionally, most studies have also reported that lower doses of oral midazolam (e.g., 0.25–0.5 mg/kg) tend to have a longer onset time compared to higher doses (e.g., 0.5–1 mg/kg) [9,23]. Higher doses of oral midazolam lead to faster sedation onset due to saturation of first-pass metabolism, resulting in higher bioavailability and more rapid systemic absorption. Additionally, increased plasma concentrations at higher doses enhance CNS penetration and GABA-A receptor activation, accelerating the sedative effect [24,25].

The procedure time recorded in this study serves as an initial indicator of the effective working duration of midazolam, assisting clinicians in treatment planning. However, it is important to acknowledge that this parameter does not precisely represent the drug’s full effective duration, as sedative effects sometimes persisted beyond procedure completion. In our study, procedure times differed significantly between 27.3 min for 0.6 mg/kg and 20.3 min for 0.3 mg/kg (*p* < 0.001). This finding aligns with prior studies demonstrating deeper sedation and longer effects at higher midazolam doses [7]. Clinicians should carefully consider the relatively short effective duration (around 20 min) of the 0.3 mg/kg dose, especially when performing more extensive procedures in anxious children, who typically present with more severe dental issues [26]. Thus, dosage selection should reflect procedure complexity, expected patient cooperation, and practical clinical considerations.

Recovery time was significantly longer in the higher-dose (0.6 mg/kg) group compared to the lower-dose (0.3 mg/kg) group (34.6 ± 4.6 min vs. 31.8 ± 4.4 min, *p* = 0.008). This finding aligns with previous studies indicating that higher midazolam doses typically result in deeper sedation and longer recovery periods [7]. Although the approximately 3 min difference between the groups was statistically significant, its clinical relevance should be carefully considered.

### 4.2. Behavior During Treatment

Patient cooperation during dental treatment was significantly better in the group receiving the higher dose of oral midazolam (0.6 mg/kg), with 95% demonstrating high cooperation compared to 78.9% in the lower-dose group (*p* = 0.045). This finding highlights a dose-dependent improvement in cooperative behavior, aligning with previous studies indicating that higher doses of oral midazolam are associated with deeper sedation and improved behavior management in pediatric dental patients [14,15,19]. Our results also showed that 8 out of 38 cases (21.1%) in the 0.3 mg/kg group could not complete treatment as initially planned due to inadequate sedation leading to patient resistance and poor cooperation.

### 4.3. Amnesic Effects

Regarding the amnesic effects, no significant differences between the two doses were observed at any evaluated time points (12 h, 24 h, and 1 week). Both groups demonstrated a similarly high rate (approximately 90%) of midazolam-induced anterograde amnesia at the 12 h assessment. This result confirms oral midazolam’s effectiveness in reducing memory recall of dental procedures, potentially contributing to decreased dental anxiety during subsequent visits. This finding is consistent with the study by Viana et al., (2017), which concluded that a higher dose of benzodiazepines did not enhance anterograde amnesia compared to a lower dose [27].

### 4.4. Safety Assessment

Regarding safety, both doses of oral midazolam (0.3 mg/kg and 0.6 mg/kg) demonstrated reassuring safety profiles in pediatric dental sedation. Throughout the procedures, heart rate and oxygen saturation (SpO_2_) remained stable and within clinically acceptable ranges in both groups. Slight decreases in heart rate observed at later time points were minimal and clinically insignificant, consistent with mild sedative effects commonly reported with midazolam use in previous studies [20,28]. Adverse events recorded were minimal and mild. Specifically, nausea was observed in 5% of patients receiving the 0.3 mg/kg dose, while mild agitation was noted in 5% of patients receiving the 0.6 mg/kg dose. These mild adverse events were infrequent and manageable without intervention.

### 4.5. Limitations

This study has several limitations that should be acknowledged. First, the sample size was relatively small, potentially limiting the statistical power and generalizability of the results. Larger-scale trials are needed to confirm these findings. Second, our trial evaluated only two specific doses of oral midazolam (0.3 mg/kg and 0.6 mg/kg), which may not represent the entire therapeutic dose range; future research exploring additional dosages would help identify the most effective and safe dosing strategies. Third, sedation levels were not continuously monitored throughout the entire procedure; implementing ongoing sedation assessments could provide more detailed insights into the sedation dynamics of oral midazolam. Finally, our study assessed sedation efficacy and amnesic effects only up to one week post-procedure. Longer-term follow-up evaluations would be valuable to better understand the sustained effects of midazolam sedation on children’s anxiety levels and cooperation in future dental visits.

## 5. Conclusions

In conclusion, our study demonstrated that oral midazolam, at both doses of 0.3 mg/kg and 0.6 mg/kg, provides effective sedation and amnesia for preschool children undergoing dental procedures. The higher dose (0.6 mg/kg) offered significantly improved patient cooperation and longer effective working duration, although it was associated with slightly prolonged recovery times. Both doses had minimal adverse events, with no severe safety concerns. Clinicians should carefully consider dose selection based on individual patient anxiety levels, procedure complexity, and practical clinical considerations.

## Figures and Tables

**Table 1:** Demographic data of the patients.

Characteristics	Group 1 (0.3 mg/kg Dose)	Group 2 (0.6 mg/kg Dose)	*p*
Age, years, mean ± SD	4.7 ± 0.9	4.7 ± 0.9	1 *
Sex, n (%)			
Male	22 (55)	21 (52.5)	0.82 **
Female	18 (45)	19 (47.5)	
Total	40 (100)	40 (100)	
ASA, n (%)			
I	32 (80)	34 (85)	0.56 **
II	8 (20)	6 (15)	
Frankl scale, n (%)			
Definitely negative	36 (90)	37 (92.5)	1 ***
Negative	4 (10)	3 (7.5)	
Weight, kg, mean ± SD	16.45 ± 2.87	16.88 ± 3.49	0.55 *

* *t*-test, ** Chi-square, *** Fisher’s exact test, SD—standard deviation.

**Table 2:** Sedation effect assessment.

Parameters	Group 1 (0.3 mg/kg Dose)	Group 2 (0.6 mg/kg Dose)	*p*-Value
Successful sedation, n (%)	38 (95)	40 (100)	0.49 *
Sedation onset time, min, mean ± SD	21.1 ± 3.1	20.3 ± 3.1	0.26 **
Dental procedure time, min, mean ± SD	20.3 ± 4.0	27.3 ± 4.1	<0.001 **
Recovery time, min, mean ± SD	31.8 ± 4.4	34.6 ± 4.6	0.008 **

* Fisher’s exact test, ** *t*-test, SD—standard deviation.

**Table 3:** During-treatment behavior assessment.

Study Classification	Houpt Scale	Group 1 (0.3 mg/kg Dose)	Group 2 (0.6 mg/kg Dose)	*p*-Value
Low Cooperation, n (%)	1-Aborted	0 (0)	0 (0)	0.045 *
	2-Poor	8 (21.1)	2 (5)	
	Total	8 (21.1)	2 (5)	
High Cooperation, n (%)	Fair	7 (18.4)	2 (5)	
	Good	12 (31.6)	11 (27.5)	
	Very good	3 (7.9)	19 (47.5)	
	Excellent	8 (21.1)	6 (15)	
	Total	30 (78.9)	38 (95)	

* Fisher’s exact test.

**Table 4:** Amnesic effect and adverse events.

Characteristics	Group 1 (0.3 mg/kg Dose)	Group 2 (0.6 mg/kg Dose)	*p*-Value
Amnesia			
After 12 h, n (%)	34 (89.5)	37 (92.5)	0.71 *
After 24 h, n (%)	36 (94.7)	40 (100)	0.231 *
After 1 week, n (%)	38 (100)	40 (100)	1 *
Adverse events			
Nausea, n (%)	2 (5)	0	NA
Agitation, n (%)	0 (0)	2 (5)	

* Fisher’s exact test, NA—not available.

**Table 5:** Vital signs.

		T0	T1	T2	T3	T4	T5
Heart rate	Group 1	117.9	117.5	101.4	101.5	101.7	104
	Group 2	118.8	117.3	104.2	104.0	99.9	99.9
SpO_2_ (%)	Group 1	98.8	96.0	96.5	97.4	97.8	97.5
	Group 2	98.6	95.6	96.5	97.4	97.7	97.4

## Data Availability

The raw data supporting the conclusions of this article will be made available by the authors on request.

## Supplementary Materials

The following supporting information can be downloaded at: https://www.mdpi.com/article/10.3390/dj13070308/s1, Table S1: The Ramsay Sedation Scale; Table S2: Houpt Behavior Rating Scale.
