# Supplementary material for: Efficacy of Oral Midazolam for Sedation and Amnesia in Preschool Children with Dental Anxiety: A Double-Blind, Randomized Controlled Trial
Source: Dent J (Basel). 2025 Jul 9;13(7):308. doi: 10.3390/dj13070308 (PMC12293258; doi:10.3390/dj13070308)
Supplement: Supplementary file 1 [file dentistry-13-00308-s001.zip › Supplementary table.pdf]

Supplementary table

Table S1: The Ramsay Sedation Scale

Score	Description
1	Awake; agitated or restless or both
2	Awake; cooperative, oriented, and tranquil
3	Awake but responds to commands only
4	Asleep; brisk response to light glabellar tap or loud auditory stimulus
5	Asleep; sluggish response to light glabellar tap or loud auditory stimulus
6	Asleep; no response to glabellar tap or loud auditory stimulus

Table S2: Houpt Behavior Rating Scale

Houpt Rating	Description
1-Aborted	Treatment cannot proceed due to excessive resistance.
2-Poor	Moderate to strong resistance, difficult to manage.
3-Fair	Occasional resistance but manageable.
4-Good	Some movement accepts treatment with minimal caution.
5-Very good	Minimal movement, cooperative, quiet.
6-Excellent	Fully cooperative, no movement, positive behavior
